# Intravitreal aflibercept for the treatment of patients with diabetic macular edema in routine clinical practice in Latin America: the AQUILA study

**DOI:** 10.1186/s40942-022-00396-y

**Published:** 2022-08-02

**Authors:** Francisco J. Rodríguez, Lihteh Wu, Arnaldo F. Bordon, Martin Charles, JinKyung Lee, Tobias Machewitz, Margarete Mueller, Gabriela del Carmen Gay, Jans Fromow-Guerra, the AQUILA Investigators, Marcelo Reinhart, Gastón Gómez Caride, Herminio Negri, Gerónimo Galván, Juan Irungaray, Mariano Irós, Matko Vidosevich, Noe Rivero, Tamara Zompa, Juan Pablo Francos, Paula Salgado, Gerardo Caceres Barrios, Octavio Regnasco, Francisco J. Rodríguez, Hildegard Piñeros, Juan Arias, Javier Buendia, Gustavo Adolfo Navarro Naranjo, Beatriz Endo, Myrian Hernandez, Lihteh Wu, Teodoro Evans Tinoco, Gerardo Garcia, Andres Padilla, Adriana Gómez Cespedes, Jose Dalma, Rene Cano, Jans Fromow-Guerra, Natalia Saldaña, Juan Manuel Jimenez, Renata del Carmen García Franco, Adriana Solis Vivanco, Angeles Yael Hernandez Vazquez

**Affiliations:** 1 Fundación Oftalmológíca Nacional, Universidad del Rosario School of Medicine, Cl. 50 ##13-50, 110231 Bogotá, DC Colombia; 2 Asociados de Macula, Vitreo y Retina de Costa Rica, Primer Piso Torre Mercedes, Paseo Colon, San José, 10102 Costa Rica; 3 Hospital Oftalmológico de Sorocaba, R. Nabek Shiroma, 210-Jarim Emilia, Sorocaba, SP 18031-060 Brazil; 4 Centro Oftalmológico Dr Charles, Riobamba 841, C116 ABA Buenos Aires, Argentina; 5 Bayer AG, 13342 Berlin, Germany; 6 Bayer SA, Doctor Ricardo Gutiérrez 3652, Munro, B1605EHD Buenos Aires, Argentina; 7 Macula Retina Consultores, Calle Sur 132, Las Américas, Álvaro Obregón, 01120 Mexico City, Mexico

**Keywords:** Macula, Vision, Clinical trial

## Abstract

**Background:**

AQUILA (NCT03470103) was a prospective, observational, 12-month cohort study to understand treatment patterns and to evaluate the clinical effectiveness and safety of intravitreal aflibercept (IVT-AFL) in patients from Latin America with diabetic macular edema (DME).

**Methods:**

Treatment-naïve and previously treated (switching to IVT-AFL) patients (aged ≥ 18 years) were enrolled from March 2018, with a primary completion date of September 2020, from Argentina, Colombia, Costa Rica and Mexico. Patients received IVT-AFL in a routine clinical practice setting.

**Results:**

Of 258 patients in the full analysis set, 181 were treatment-naïve and 77 had received previous treatment. The mean ± standard deviation number of IVT-AFL injections by Month 12 was 3.7 ± 1.8 (treatment-naïve) and 4.0 ± 2.2 (previously treated). The median duration from diagnosis to IVT-AFL treatment was 1.8 months (treatment-naïve) and 16.0 months (previously treated). Mean best-corrected visual acuity (Early Treatment Diabetic Retinopathy Study letters) improved from baseline to Month 12 by + 8.1 ± 17.7 (treatment-naïve; baseline: 54.5 ± 19.4) and + 4.6 ± 15.4 letters (previously treated; baseline: 52.9 ± 18.6).

**Conclusion:**

AQUILA is the first study to assess the use of IVT-AFL in routine clinical practice in Latin America. Despite few patients being treated with the label-recommended regimen of 5 initial monthly doses or receiving ≥ 8 injections in 12 months, functional and anatomic visual outcomes improved during 12 months of treatment with IVT-AFL. Patients receiving the label-recommended number of injections had numerically greater improvements in visual acuity outcomes. Patients with DME treated regularly and more frequently with IVT-AFL therefore have the potential to achieve outcomes consistent with those observed in interventional studies.

*Trial registration* Clinicaltrials.gov, NCT03470103. Registered February 5, 2018, https://clinicaltrials.gov/ct2/show/NCT03470103

**Supplementary Information:**

The online version contains supplementary material available at 10.1186/s40942-022-00396-y.

## Background

An estimated 31.6 million people were estimated to be living with diabetes in Latin America in 2019 [1]. The incidence of diabetes is predicted to increase (49.1 million people in Latin America are estimated to have diabetes by 2045) [1], and the prevalence of diabetic retinopathy (DR) is also increasing [2–4] (15–85% of Latin American patients with diabetes have some degree of DR, with the prevalence of DR being higher in more developed countries) [5]. Diabetic macular edema (DME) is a frequently occurring sub-type of DR, and arises in 3–10% of patients with diabetes [5]. DME is expected to increase in prevalence, due to increased life expectancy resulting from improving socioeconomic and sociodemographic status in Latin America [1, 3], as well as other factors. Despite improvements in screening program and systemic treatment options for patients with diabetes, this increase in DR and DME incidence is still expected to occur globally.

Current treatment guidelines for DME in Latin America include the use of anti-vascular endothelial growth factor (anti-VEGF) therapies, laser therapy, intravitreal steroid injections, and intravitreal steroid implants [5], in addition to dietary and lifestyle changes for improved glycemic control.

The anti-VEGF therapies aflibercept and ranibizumab are licensed for use in DME in Latin America, and bevacizumab is used off-label at the discretion of the prescribing physician [5]. Intravitreal aflibercept (IVT-AFL) was approved following the VIVID [6, 7] and VISTA [6, 8] clinical trials, which demonstrated superiority of IVT-AFL over laser therapy in both visual and anatomical outcomes. The data derived from VIVID and VISTA led to the approval of IVT-AFL in Argentina, Colombia, Costa Rica, and Mexico, and anti-VEGF therapies are considered to be the gold standard for DME care in Latin America and globally.

Although many Latin American countries have been addressing the increasing prevalence of DME (for example, by initiating referral networks) [9], observational data on the impact of anti-VEGF treatment on DME outcomes in routine clinical practice are lacking. Observational studies provide real-world evidence (RWE) that is complementary to data derived from randomized controlled trials regarding the effectiveness of a therapy outside of a controlled environment. This valuable RWE on therapy effectiveness enables better clinical decision-making across ophthalmology and provides useful insights into opportunities for optimization in clinical practice [10, 11].

AQUILA was a prospective observational cohort study in patients with DME or neovascular age-related macular degeneration (nAMD) designed to **A**ssess the fre**Q**uency of **U**se of **I**VT-AFL in routine clinical practices in **L**atin **A**merica (NCT03470103). The aim of this manuscript is to evaluate the clinical effectiveness, safety, and treatment patterns of IVT-AFL in DME in routine clinical practice in Latin America in both treatment-naïve and previously treated (switched to IVT-AFL) patients.

## Methods

### Study design and treatment

The AQUILA study (NCT03470103) was conducted in accordance with the Declaration of Helsinki and the International Council for Harmonisation guideline E6: Good Clinical Practice. The protocol and any amendments were reviewed and approved by each study site’s Independent Ethics Committee or Institutional Review Board before the start of the study. AQUILA enrolled treatment-naïve and previously treated patients with DME (aged ≥ 18 years) or nAMD (aged ≥ 55 years) from March 2018, with a primary completion date of September 2020. Treatment-naïve patients had not received previous intravitreal treatment, including anti-VEGF agents, steroids, and steroid implants. Previously treated patients had received a different treatment (for example, anti-VEGF therapy or steroids) and were switching to IVT-AFL. Patients received IVT-AFL treatment according to decisions made at the discretion of the prescribing physician, according to their medical practice. The results of patients with nAMD are reported separately.

### Participants

Patients were enrolled from 13 clinics in Argentina, seven clinics in Colombia, two clinics in Costa Rica, and 11 clinics in Mexico. Patients became eligible for AQUILA once the decision was made to treat with IVT-AFL (either receiving anti-VEGF therapy for the first time or switching from a different anti-VEGF therapy to IVT-AFL). The prescribing information for IVT-AFL recommends a treatment regimen of 5 initial monthly injections of 2 mg followed by an injection every 2 months [12].

Key exclusion criteria included patients participating in a current clinical trial outside of routine practice, patients currently receiving IVT-AFL or another anti-VEGF agent for their disease, patients receiving an anti-VEGF other than IVT-AFL in the fellow eye, patients receiving concomitant ocular or systemic administration drugs that could affect the mechanism of IVT-AFL, or patients with ocular or peri-ocular infections in either eye, or active intraocular inflammation/scar/fibrosis/atrophy/advanced glaucoma/cataracts in the study eye.

### Study endpoints and analysis

The primary efficacy endpoint was change from baseline to Month 12 in best-corrected visual acuity (BCVA; Early Treatment Diabetic Retinopathy Study [ETDRS] letters). Secondary endpoints included: treatment patterns at Month 12 (number of injections/monitoring/combined visits, number of visual acuity [VA] tests, number of fundoscopy examinations, and number of optical coherence tomography [OCT] assessments); duration and type of previous treatments and reason for switch to IVT-AFL in previously treated patients; mean time between IVT-AFL injections and mean number of IVT-AFL injections at Month 12; duration and type of previous treatments and reason for switch to IVT-AFL (previously treated only); number of patients achieving a Snellen equivalent of 20/40 or better (~ 70 ETDRS letters) at Month 12; number of patients gaining ≥ 15 ETDRS letters at Month 12; change from baseline to Month 12 in central retinal thickness (CRT); and number of patients with no fluid determined by OCT at Month 12.

Patients who received at least one IVT-AFL injection were included in the safety analysis set (SAF). Patients were included in the full analysis set (FAS) if they received at least one IVT-AFL injection and had a BCVA assessment in the study eye at both baseline and at least one follow-up visit. Data were analyzed descriptively. Selected continuous variables were categorized prior to study initiation in a clinically meaningful way for analysis (VA at baseline, number of injections within 6/12 months). Last observation carried forward (LOCF) was used to impute missing values for BCVA and CRT measurements. Missing values for other variables (for example, fluid) were not imputed.

## Results

### Baseline demographics and disease characteristics

Of the 330 patients screened for inclusion in this study, 11 did not receive treatment and were not included in the SAF. Of 319 patients in the SAF, 61 patients did not have a valid BCVA letter score at baseline, or post-baseline, and thus were ineligible for inclusion in the FAS; the overall FAS therefore comprised 258 patients (Additional file 1: Fig. S1).

Table 1 depicts patient baseline demographics and disease characteristics. The most frequently reported comorbidities (ongoing/having recovered from) were hypertension (49.6% of patients), cataracts (20.5% of patients), and hyperlipidemia (9.7% of patients). The mean ± standard deviation (SD) BCVA letter score in the study eye was 54.0 ± 19.2 (approximately 20/80 Snellen).

**Table 1:** Patient baseline demographics and disease characteristics (FAS)

	Treatment-naïve (n = 181)	Previously treated (n = 77)	Overall (n = 258)
Age, years, mean ± SD	64.6 ± 9.8	63.0 ± 8.6	64.1 ± 9.5
Female	70 (38.7)	43 (55.8)	113 (43.8)
Country			
Argentina	111 (61.3)	25 (32.5)	136 (52.7)
Colombia	11 (6.1)	7 (9.1)	18 (7.0)
Costa Rica	3 (1.7)	6 (7.8)	9 (3.5)
Mexico	56 (30.9)	39 (50.7)	95 (36.8)
Diabetes mellitus			
Type 1	18 (9.9)	0	18 (7.0)
Type 2	163 (90.1)	77 (100)	240 (93.0)
Severity of diabetic retinopathy			
Mild	26 (14.4)	10 (13.0)	36 (14.0)
Moderate	66 (36.5)	23 (29.9)	89 (34.5)
Severe	76 (42.0)	40 (52.0)	116 (45.0)
Missing	13 (7.2)	4 (5.2)	17 (6.6)
Comorbidities^a^			
Hypertension	76 (42.0)	52 (67.5)	128 (49.6)
Cataracts	34 (18.8)	19 (24.7)	53 (20.5)
Hyperlipidemia	18 (9.9)	7 (9.1)	25 (9.7)
Obesity	8 (4.4)	11 (14.3)	19 (7.4)
BCVA in the study eye, mean ± SD letter score	54.5 ± 19.4	52.9 ± 18.6	54.0 ± 19.2
Categorical BCVA letter score, n (%)			
≥ 70 letters (≥ 20/40 Snellen)	50 (27.6)	17 (22.1)	67 (26.0)
< 70 letters (< 20/40 Snellen)	131 (72.4)	60 (77.9)	191 (74.0)
CRT, μm, mean ± SD	388 ± 145	423 ± 146	398 ± 146

*BCVA* best-corrected visual acuity, *CRT* central retinal thickness, *FAS* full analysis set, *SD* standard deviation

^a^Reported in ≥ 5% of patients. Values are n (%) unless otherwise stated

A total of 181 patients were treatment-naïve and 77 patients were previously treated: with ranibizumab without focal laser (n = 53, 68.8%), bevacizumab without focal laser (n = 17, 22.1%), ranibizumab/bevacizumab with focal laser (n = 4, 5.2%), ranibizumab/bevacizumab with focal laser and steroids (n = 3, 3.9%), or steroids without focal laser (n = 2, 2.6%). Additional file 1: Table S1 contains the duration of previous treatment for DME (previously treated, FAS), and reasons for switch from a previous treatment to IVT-AFL.

In treatment-naïve patients, the median time from diagnosis of DME to first injection of IVT-AFL was 1.8 months (interquartile range [IQR] 0.4–4.5). In previously treated patients who had already received a mean of 13.4 months of anti-VEGF treatment (ranibizumab or bevacizumab), the median time from diagnosis of DME to first injection of IVT-AFL was 16.0 months (IQR 8.8–32.8).

### Treatment regimens and visits

Planned treatment regimens for patients reflected the reported treatment regimens (Table 2). The number of IVT-AFL injections received by patients is reported in Table 2; the mean dosing interval time (after the first 180 days) was 39.2 days (IQR 24.5–49.0). The number of clinical visits for injections, monitoring visits without injections, and combined visits for injections and monitoring are shown in Additional file 1: Table S2. Most patients had 1–3 clinical visits, 1–3 monitoring visits, and 1–3 combined visits.

**Table 2:** Injections and planned/observed treatment regimen (FAS)

	Treatment-naïve (n = 181)	Previously treated (n = 77)	Overall (n = 258)
Planned treatment regimen			
T&E from initial treatment	29 (16.0)	10 (13.0)	39 (15.1)
5 initial monthly injections followed by T&E	39 (21.6)	18 (23.4)	57 (22.1)
5 initial monthly injections followed by injections every other month	4 (2.2)	0	4 (1.6)
Treat until dry followed by T&E	20 (11.1)	11 (14.3)	31 (12.0)
Treat until dry followed by PRN	42 (23.2)	13 (16.9)	55 (21.3)
PRN from initial treatment	28 (15.5)	19 (24.7)	47 (18.2)
Other	19 (10.5)	6 (7.8)	25 (9.7)
Reported treatment regimen^a^			
T&E	25 (13.8)	10 (13.0)	35 (13.6)
5 initial monthly injections followed by T&E	13 (7.2)	6 (7.8)	19 (7.4)
Treat until dry followed by T&E	17 (9.4)	10 (13.0)	27 (10.5)
Treat until dry followed by PRN	60 (33.2)	11 (14.3)	71 (27.5)
PRN from initial treatment	29 (16.0)	21 (27.3)	50 (19.4)
No initial treatment	10 (5.5)	7 (9.1)	17 (6.6)
Other	27 (14.9)	12 (15.6)	39 (15.1)
Mean IVT-AFL injections by Month 6 (mean ± SD)	2.9 ± 1.2	3.2 ± 1.6	3.0 ± 1.3
Mean IVT-AFL injections by Month 12 (mean ± SD)	3.7 ± 1.8	4.0 ± 2.2	3.8 ± 1.9
≥ 5 injections within 6 months	13 (7.2)	19 (24.7)	32 (12.4)
≥ 8 injections within 12 months	7 (3.9)	7 (9.1)	14 (5.4)

Data are n (%), unless otherwise stated

*FAS* full analysis set, *IVT-AFL* intravitreal aflibercept, *PRN* *pro re nata*, *SD* standard deviation, *T&E* treat and extend

^a^As reported by the investigator(s)

### Functional and anatomic outcomes

**Fig. 1:**
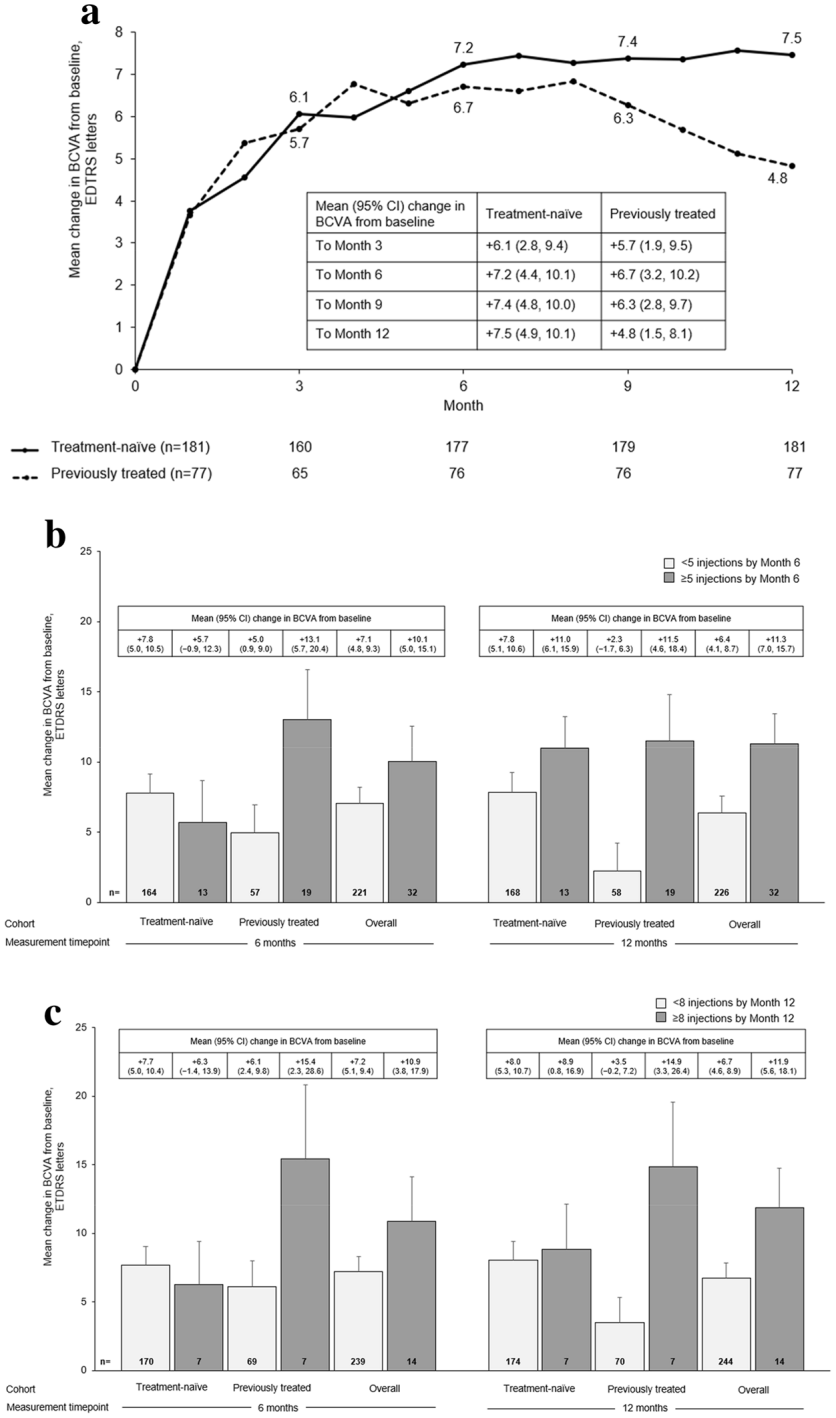

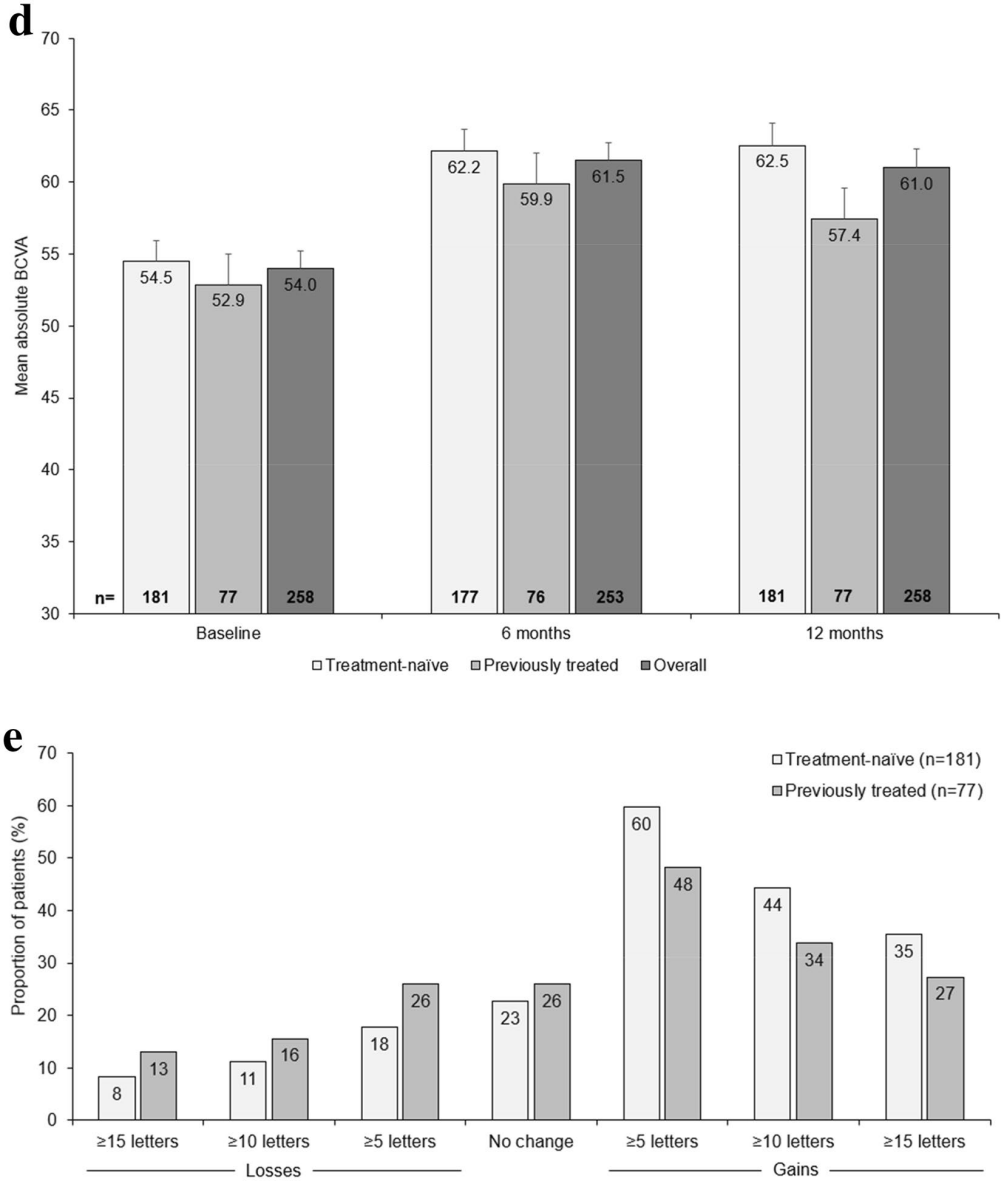
Visual acuity outcomes (FAS). **a** Mean change in BCVA letter score over 12 months in treatment-naïve and previously treated patients; **b** Mean change in BCVA letter score at Months 6 and 12 in treatment-naïve and previously treated patients by number of injections (< 5 or ≥ 5) received in the first 6 months of treatment; **c** Mean change in BCVA letter score at Months 6 and 12 in treatment-naïve and previously treated patients by number of injections received overall; **d** Mean absolute BCVA letter score at Months 6 and 12 in treatment-naïve and previously treated patients; **e** Proportion of treatment-naïve and previously treated patients by BCVA categorical score change. Missing data imputed using LOCF. Data reported as mean ± SE, where relevant. Data in A were collected monthly ± 15 days; number of patients with assessment at the indicated timepoint indicated below figure. Data in figures B–D, for the 6-month timepoint was collected at 6 months ± 30 days; data for the 12-month timepoint was collected at 12 months ± 60 days. *BCVA* best-corrected visual acuity, *ETDRS* Early Treatment Diabetic Retinopathy Study, *FAS* full analysis set, *LOCF* last observation carried forward, *SE* standard error

An improvement in BCVA over 12 months (the primary endpoint) was observed in both patient groups; the increase in BCVA ± SD [95% confidence interval, CI] at 12 months was numerically higher in treatment-naïve patients (+ 8.1 ± 17.7 [5.5, 10.7] letters) than in previously treated patients (+ 4.6 ± 15.4 [1.0, 8.1] letters). Mean change in BCVA from baseline to Month 12 for treatment-naïve and previously treated patients is shown in Fig. 1A.

Patients receiving ≥ 5 IVT-AFL injections in the initial phase of treatment (as recommended in the prescribing information) [12] achieved numerically higher gains in BCVA after 12 months of IVT-AFL treatment, regardless of prior treatment (Fig. 1B). Similarly, patients receiving the recommended ≥ 8 IVT-AFL injections also achieved numerically higher gains in BCVA after 12 months of treatment (Fig. 1C). Figure 1D depicts mean absolute BCVA letter score; gains in BCVA were marginally numerically larger in treatment-naïve patients. A higher proportion of treatment-naïve patients compared with previously treated patients had BCVA improvements of ≥ 15 letters at Month 12 (Fig. 1E); and the proportion of patients with a loss of ≥ 15 letters at Month 12 was higher in the previously treated cohort than in the treatment-naïve cohort. The proportion of patients with a BCVA of ≥ 70 letters increased from 27.6% at baseline to 50.8% at Month 12 in treatment-naïve patients, and from 22.1% at baseline to 32.5% at Month 12 in previously treated patients.

**Fig. 2:**
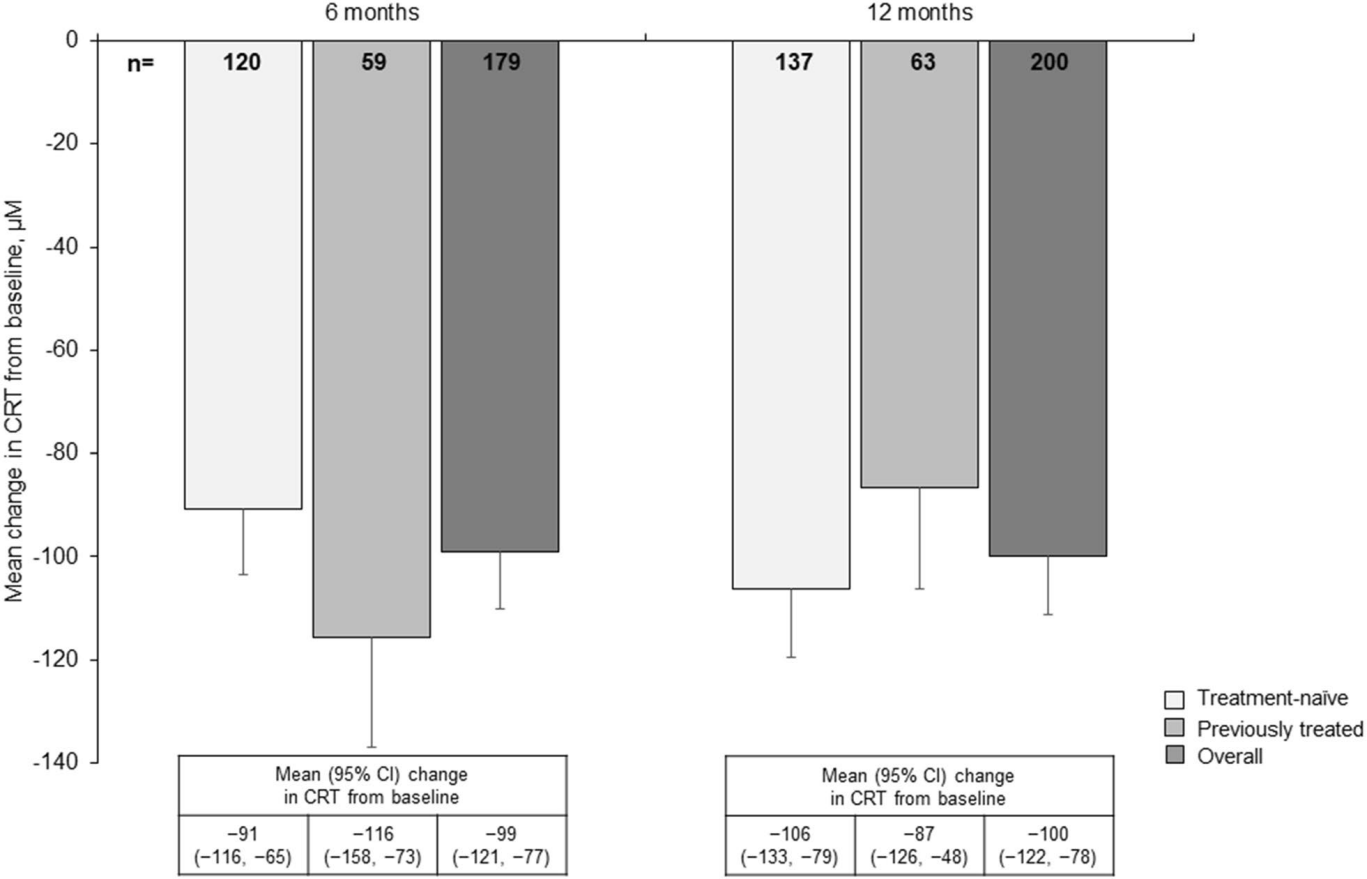
Mean change in CRT over 12 months in treatment-naïve and previously treated patients. Data reported as mean ± SE. Missing data imputed using LOCF. *CRT* central retinal thickness, *LOCF* last observation carried forward, *SE* standard error

Figure 2 depicts mean change in CRT from baseline to 12 months. The proportion of patients with intraretinal fluid, subretinal fluid and subretinal pigment epithelium fluid at baseline and after 12 months of treatment with IVT-AFL is shown in Additional file 1: Fig. S2. The proportion of patients without any fluid increased from 3% at baseline to 16% at Month 12; however, data were missing at Month 12 for 59% of patients. Five patients were unable to complete 12 months due to COVID-19, and the mean change in BCVA (95% CI) up to 12 months was + 8.1 (5.3, 10.9) letters in patients treated pre-COVID-19 (n = 147) and + 5.6 (2.4, 8.8) letters in patients treated during the pandemic (n = 111).

### Safety

An overview of the main safety data is shown in Table 3. There were no cases of endophthalmitis or retinal vasculitis, and 1 case of iridocyclitis, which was deemed not to be serious by the investigator. Six treatment-related events were reported; all were ocular-related (1 incidence each of: cataracts, iridocyclitis, ocular hypertension, vitreous hemorrhage, bacterial conjunctivitis, and unspecified conjunctivitis). Serious ocular adverse events (AEs) occurring in 6 patients were: cataracts, worsening of DR, glaucoma, retinal artery occlusion and visual impairment (1 case each), and vitreous hemorrhage (2 cases). One of these serious ocular events was deemed to be treatment-related by the investigator (cataracts). Seven deaths were reported during the 12-month study: multiorgan failure, heart failure, acute myocardial infarction, cardiorespiratory failure (1 death each; all considered to be unrelated to IVT-AFL); the cause of death was unknown in 3 patients.

**Table 3:** Safety overview (SAF)

Number of patients (%)	Safety analysis set N = 319
Any AE^a^	42 (13.2)
Ocular AEs^b^	30 (9.4)
Vitreous hemorrhage	8 (2.5)
Worsening of diabetic retinopathy	6 (1.9)
Cataract	3 (0.9)
Glaucoma	3 (0.9)
Treatment-related ocular AEs	6 (1.9)
Serious ocular AEs	6 (1.9)
Treatment-related serious ocular AEs	1 (0.3)
Non-ocular AEs	16 (5.0)
Treatment-related non-ocular AEs	0
Serious non-ocular AEs^c^	12 (3.8)
Deaths^d^	7 (2.2)

*AE* adverse event, *IVT-AFL* intravitreal aflibercept, *SAF* safety analysis set

^a^AEs are those reported if they started after the first IVT-AFL injection and not later than 30 days after the last IVT-AFL injection. If no unambiguous allocation is possible because of missing parts of the AE start date, for example, the AE will be treated as an AE (worst case scenario)

^b^Ocular AEs reported by preferred term in ≥ 3 patients

^c^Deemed to be unrelated to treatment according to the responsible physician

^d^The cause of death was unknown for 3 patients; and the 4 other deaths were deemed to be unrelated to treatment according to the responsible physician

## Discussion

AQUILA is the first study to assess the use of IVT-AFL in routine clinical practice in Latin America, and one of the first observational, real-world studies of anti-VEGF agents in Latin America. Treatment-naïve and previously treated patients receiving IVT-AFL prescribed according to their treating physician experienced improvements in functional and anatomical outcomes after 12 months of treatment. By Month 12, the mean improvement in BCVA was + 8.1 letters in treatment-naïve patients and + 4.6 letters in previously treated patients; CRT decreased by 106 μm and 87 μm in treatment-naïve and previously treated patients, respectively. Improvements in BCVA were evident by Month 6 in both treatment-naïve and previously treated patients and maintained through to Month 12 in treatment-naïve patients. Differences in BCVA achievement between treatment-naïve patients and previously treated patients may be explained by the previously treated patients achieving some level of improvement in VA with their previous treatment before enrolling in AQUILA; or that their previous treatment was ineffective/inappropriate if they lost VA during their previous treatment period.

Patients receiving the recommended number of IVT-AFL injections (according to the labelling information) had numerically better results than those receiving fewer than the recommended number of injections. A higher proportion of patients received fewer than the recommended number of IVT-AFL injections during AQUILA, which is consistent with other observational studies of IVT-AFL [13] and other anti-VEGF therapies for DME [14]. In routine clinical practice, patients with DME undergo fewer anti-VEGF injections and exhibit reduced visual gains than patients in randomized controlled trials [15]. Although patients were under-treated according to the labelling information [12], the improvements observed indicate that the magnitude of the effects could be larger if regimens more closely aligned to those recommended.

RWE of anti-VEGF treatment for DME in Latin America was provided by LUMINOUS, a global, prospective, observational study of ranibizumab, and included treatment-naïve patients from Latin America (including Argentina, Colombia, Costa Rica, and Mexico) [14]. The mean number of injections over the first year of LUMINOUS was 4.5, compared to 3.8 injections in AQUILA. A total of 47.5% of patients received ≥ 5 injections, compared to 29.5% of patients in AQUILA. The labelling information for ranibizumab recommends more frequent injections than the labelling information for IVT-AFL (once per month) [12, 16]; however, the injection numbers reported in LUMINOUS were substantially lower than expected and consistent with the injection numbers reported in AQUILA.

The Pan-American Collaborative Retina Study Group (PACORES) provided 5-year results from a Latin American retrospective study of bevacizumab [17, 18], which indicated statistically significant improvement in visual outcomes over the first 3 years. Patients received few injections (8.4 ± 7.1) over 5 years, providing further evidence of undertreatment during routine clinical practice in Latin America.

In APOLLON [13], a prospective, observational cohort study of treatment-naïve and previously treated French patients with DME who were treated with IVT-AFL, patients received an average of 7.6 injections, compared to 3.8 injections in AQUILA. Despite this, BCVA outcomes were similar: in APOLLON, mean change in BCVA at Month 12 was + 7.8 letters in treatment-naïve patients and + 5.0 in previously treated patients. Similarly, 45.5% of treatment-naïve patients in APOLLON achieved a gain of ≥ 10 letters, compared to 44.2% of treatment-naïve patients in AQUILA.

In the first year of Protocol T (a United States-based randomized clinical trial comparing IVT-AFL, ranibizumab and bevacizumab on a fixed-treatment regimen) [19], patients received 9.2, 9.4, and 9.7 injections of IVT-AFL, ranibizumab, and bevacizumab, respectively. Mean improvement in BCVA letter score was + 13.3, + 11.2, and + 9.7 letters, for IVT-AFL, ranibizumab, and bevacizumab, respectively. Two-year data from Protocol T [20] showed that patients either maintained their VA gains, or improved, despite patients receiving numerically fewer injections over the second year (5.0, 5.4, and 5.5 injections of IVT-AFL, ranibizumab, and bevacizumab, respectively). Despite patients in AQUILA not receiving the recommended number of injections over 12 months, BCVA achievements were in line with previous research; however, if patients were to receive the number of IVT-AFL injections recommended by the labelling information, improvements in visual outcomes may be in line with those observed in phase 3 studies.

The reasons for not achieving the optimal IVT-AFL injection numbers were not captured during AQUILA, thus the data should be interpreted through the lens of the healthcare systems in Latin America. Argentina has more than 600 different healthcare insurance providers, and it is not mandatory to reimburse for treatment with anti-VEGFs; reimbursement is dependent on the payer, which affects how much treatment patients are willing or able to pay for. Patients in Colombia receive their healthcare from a publicly funded insurance, which may only cover partial reimbursement of injections, potentially limiting the capacity for treatment. The Costa Rican national healthcare system is funded by taxpayers through employment taxes, with the individual and employer contributing towards healthcare; bevacizumab is the only anti-VEGF agent available via the national healthcare system. Patients may seek private physicians via private insurance, if they can afford to do so. Private insurance covers IVT-AFL, so most private patients will pay for their anti-VEGF treatment. Patients enrolled in AQUILA from Mexico paid for their own treatment; the few patients who had private insurance policies were often reimbursed.

The safety profile of IVT-AFL during AQUILA was consistent with previous studies; no new safety concerns were observed. The incidence of AEs of interest is consistent with safety data from previous phase 3 clinical trials [6, 19] and observational studies [13].

One possible limitation of AQUILA was the reliance on BCVA as the key efficacy parameter; 59% of patients do not have fluid data at Month 12, and CRT data are missing for approximately 22% of patients. This could be due to country-specific reimbursement limitations for OCT testing, indicating that treatment of DME involves factors outside of the medicine itself. There is, therefore, room for improvement in the care of such patients. The study population predominantly comes from Argentina, and thus introduces population bias, with the Argentinian healthcare system insurance largely influencing the treatment pattern data and availability of OCT measurements. The BCVA effects for the patients observed with a certain number of injections, and those without, are confounded due to the number of injections being determined post-baseline, which can be considered an outcome itself. Any interpretations must consider their relationship as associative, rather than causative. Additionally, patients lost to follow-up in this study were imputed using LOCF, which may have further skewed results from patients who may not have been able to continue treatment.

## Conclusions

AQUILA is the first study to assess the use of IVT-AFL in routine clinical practice in Latin American patients with DME. In AQUILA, despite few patients having received the recommended regimen of 5 initial monthly doses or ≥ 8 injections in 12 months, functional and anatomical outcomes still improved during 12 months of treatment with IVT-AFL. Numerically greater improvements in functional and anatomical outcomes were observed in treatment-naïve patients compared to previously treated patients, and in patients who received ≥ 5 initial monthly injections compared to those who did not. Thus, in real-world studies, patients with DME treated regularly and more frequently with IVT-AFL have the potential to achieve outcomes that are consistent with those observed in interventional studies.

## Supplementary Information

**Additional file 1: Table S1.** Duration of previous treatment for DME (previously treated, FAS), and reasons for switch to IVT-AFL. **Table S2.** Proportion of patients with no, 1–3, 4–6, 7–9 and ≥10 clinical, monitoring or combined visits by Month 12, and proportion of patients with a non-ophthalmology visit by Month 12 (FAS). **Fig. S1.** Patient disposition (CONSORT flow diagram). **Fig. S2.** Fluid status at (a) baseline, (b) Month 6, and (c) Month 12, in treatment-naïve and previously treated patients. **Appendix**. List of participating investigators and clinics.

## Data Availability

Availability of the data underlying this publication will be determined according to Bayer’s commitment to the EFPIA/PhRMA “Principles for responsible clinical trial data sharing”. This pertains to scope, time point, and process of data access. As such, Bayer commits to sharing upon request from qualified scientific and medical researchers patient-level clinical trial data, study-level clinical trial data, and protocols from clinical trials in patients for medicines and indications approved in the United States (US) and European Union (EU) as necessary for conducting legitimate research. This applies to data on new medicines and indications that have been approved by the EU and US regulatory agencies on or after January 01, 2014. Interested researchers can use www.clinicalstudydatarequest.com to request access to anonymized patient-level data and supporting documents from clinical studies to conduct further research that can help to advance medical science or improve patient care. Information on the Bayer criteria for listing studies and other relevant information is provided in the Study sponsors section of the portal. Data access will be granted to anonymized patient-level data, protocols, and clinical study reports after approval by an independent scientific review panel. Bayer is not involved in the decisions made by the independent review panel. Bayer will take all necessary measures to ensure that patient privacy is safeguarded.

## Abbreviations

AE: Adverse event
Anti-VEGF: Anti-vascular endothelial growth factor
BCVA: Best-corrected visual acuity
CRT: Central retinal thickness
DME: Diabetic macular edema
DR: Diabetic retinopathy
ETDRS: Early Treatment Diabetic Retinopathy Study
FAS: Full analysis set
IQR: Interquartile range
IVT-AFL: Intravitreal aflibercept
LOCF: Last observation carried forward
nAMD: Neovascular age-related macular degeneration
OCT: Optical coherence tomography
PRN: PRN, pro re nata
RWE: Real-world evidence
SAF: Safety analysis set
T&E: Treat and extend
VA: Visual acuity
