# Supplementary material for: Intravitreal aflibercept for the treatment of patients with diabetic macular edema in routine clinical practice in Latin America: the AQUILA study
Source: Int J Retina Vitreous. 2022 Aug 2;8:52. doi: 10.1186/s40942-022-00396-y (PMC9344444; doi:10.1186/s40942-022-00396-y)
Supplement: Supplementary file 1 — Additional file 1: Table S1. Duration of previous treatment for DME (previously treated, FAS), and reasons for switch to IVT-AFL. Table S2. Proportion of patients with no, 1–3, 4–6, 7–9 and ≥10 clinical, monitoring or combined visits by Month 12, and proportion of patients with a non-ophthalmology visit by Month 12 (FAS). Fig. S1. Patient disposition (CONSORT flow diagram). Fig. S2. Fluid status at (a) baseline, (b) Month 6, and (c) Month 12, in treatment-naïve and previously treated patients. Appendix. List of participating investigators and clinics. [file 40942_2022_396_MOESM1_ESM.docx]

**Additional content**

**9 July 2022**

# Title

Intravitreal aflibercept for the treatment of patients with diabetic macular edema in routine clinical practice in Latin America: The AQUILA study

**Authors:**

Francisco J. Rodríguez,^1^ Lihteh Wu,^2^ Arnaldo F. Bordon,^3^ Martin Charles,^4^ JinKyung Lee,^5^ Tobias Machewitz,^5^ Margarete Mueller,^5^ Gabriela del Carmen Gay,^6^ Jans Fromow-Guerra,^7^ on behalf of the AQUILA investigators

**Affiliations:**^1^Fundación Oftalmológíca Nacional, Universidad del Rosario School of Medicine, Bogotá, Colombia
^2^Asociados de Macula, Vitreo y Retina de Costa Rica, San José, Costa Rica
^3^Hospital Oftalmológico de Sorocaba, Sorocaba, Brazil
^4^Centro Oftalmológico Dr Charles, Buenos Aires, Argentina
^5^Bayer AG, Berlin, Germany
^6^Bayer SA, Munro, Argentina
^7^Macula Retina Consultores, Mexico City, Mexico

**Correspondence to:**

Francisco J. Rodríguez, MD
Fundacion Oftalmológica Nacional,
Cl. 50 #13-50,
Bogota, DC, Colombia

[fjrodriguez@fon.org.co](mailto:fjrodriguez@fon.org.co)

Additional content

[1](#_Toc98427530)

[**Table S1.** Duration of previous treatment for DME (previously treated, FAS), and reasons for switch to IVT-AFL **3**](#_Toc98427531)

[**Table S2.** Proportion of patients with no, 1–3, 4–6, 7–9 and ≥10 clinical, monitoring or combined visits by Month 12, and proportion of patients with a non-ophthalmology visit by Month 12 (FAS) **4**](#_Toc98427532)

[**Fig. S1.** Patient disposition (CONSORT flow diagram) **5**](#_Toc98427533)

[**Fig. S2.** Fluid status at (**a**) baseline, (**b**) Month 6, and (**c**) Month 12, in treatment-naïve and previously treated patients **6**](#_Toc98427534)

[**Appendix.** List of participating investigators and clinics**. 7**](#_Toc98427535)

# Table S1. Duration of previous treatment for DME (previously treated, FAS), and reasons for switch to IVT-AFL

| **Time between first and last treatments in months, mean ± SD^a^** |  |
| --- | --- |
| Ranibizumab (n = 45) | 11.4 ± 12.7 |
| Bevacizumab (n = 16) | 14.6 ± 21.5 |
| Focal laser (n = 4) | 24.6 ± 17.5 |
| Triamcinolone (n = 1) | 5.3 |
| Dexamethasone (n = 1) | 20.8 |
| Total (n = 61) | 13.4 ± 16.1 |
| **Primary reason for switch to IVT-AFL, n (%)** |  |
| Persistence of fluid (intraretinal or subretinal) | 49 (63.6) |
| Recurrence of fluid | 14 (18.2) |
| New haemorrhage – bleeding | 1 (1.3) |
| Decreased vision | 3 (3.9) |
| Lack of compliance | 2 (2.6) |
| Patient request | 4 (5.2) |
| Other | 4 (5.2) |

^a^Missing n = 16. DME, diabetic macular eedema; FAS, full analysis set; IVT-AFL, intravitreal aflibercept; SD, standard deviation.

# Table S2. Proportion of patients with no, 1–3, 4–6, 7–9, and ≥10 clinical, monitoring, or combined visits by Month 12, and proportion of patients with a non-ophthalmology visit by Month 12 (FAS)

|  | **n (%)** | **Treatment-naïve (n = 181)** | **Previously treated (n = 77)** | **Overall (n = 258)** |  |
| --- | --- | --- | --- | --- | --- |
| *Clinical  visits^a^* | None | 36 (19.9) | 23 (29.9) | 59 (22.9) |  |
|  | 1–3 | 110 (60.8) | 40 (52.0) | 150 (58.1) |  |
|  | 4–6 | 31 (17.1) | 13 (16.9) | 44 (17.1) |  |
|  | 7–9 | 4 (2.2) | 1 (1.3) | 5 (1.9) |  |
|  | ≥10 | 0 | 0 | 0 |  |
| *Monitoring visits^b^* | | *None* | 4 (2.2) | 3 (3.9) | 7 (2.7) |
|  |  | 1–3 | 77 (42.5) | 35 (45.5) | 112 (43.4) |
|  |  | 4–6 | 60 (33.2) | 24 (31.2) | 84 (32.6) |
|  |  | 7–9 | 25 (13.8) | 11 (14.3) | 36 (14.0) |
|  |  | ≥10 | 15 (8.3) | 4 (5.2) | 19 (7.4) |
| *Combined  visits^c^* | | 1–3 | 170 (93.9) | 60 (77.9) | 230 (89.2) |
|  |  | 4–6 | 8 (4.4) | 11 (14.3) | 19 (7.4) |
|  |  | 7–9 | 2 (1.1) | 4 (5.2) | 6 (2.3) |
|  |  | ≥10 | 1 (0.6) | 2 (2.6) | 3 (1.2) |

^a^Clinical visit for injection; ^b^visit only for diagnostic purposes, without injections; ^c^visit for monitoring and injections. FAS, full analysis set.

# Fig. S1. Patient disposition (CONSORT flow diagram)


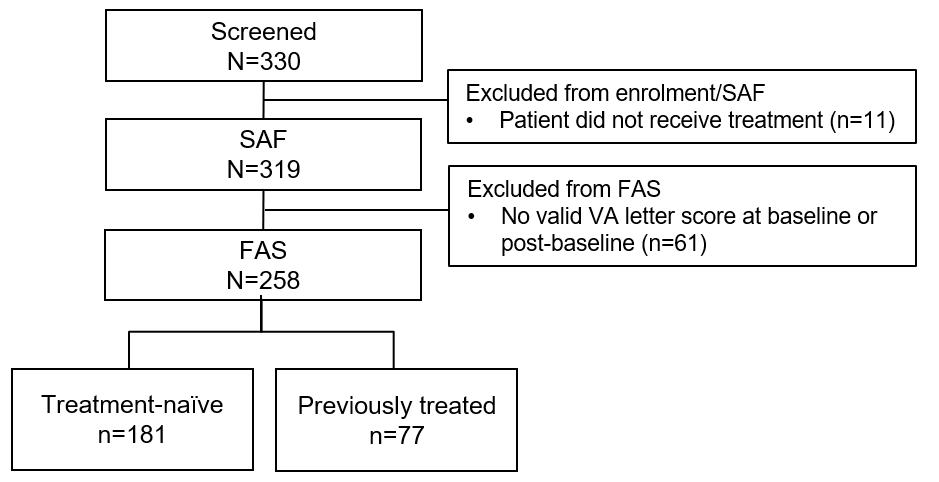
306 patients completed up to 6 months of treatment, and 216 completed up to 12 months of treatment. FAS, full analysis set; SAF, safety analysis set; VA, visual acuity.

# Fig. S2. Fluid status at (a) baseline, (b) Month 6, and (c) Month 12, in treatment-naïve and previously treated patients

**a**


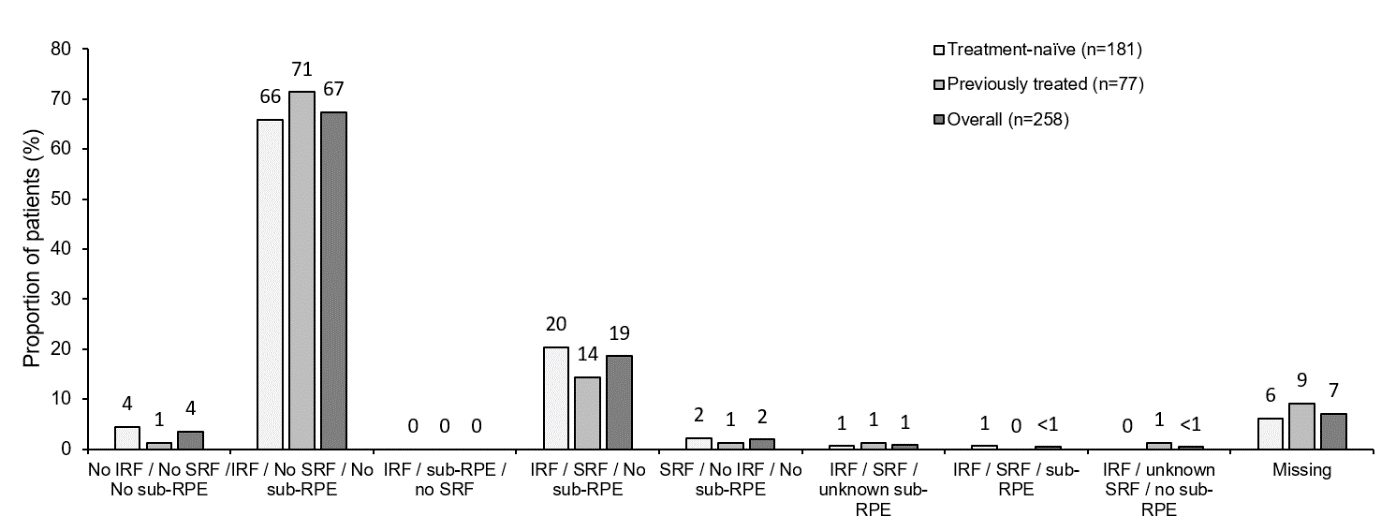


**b**


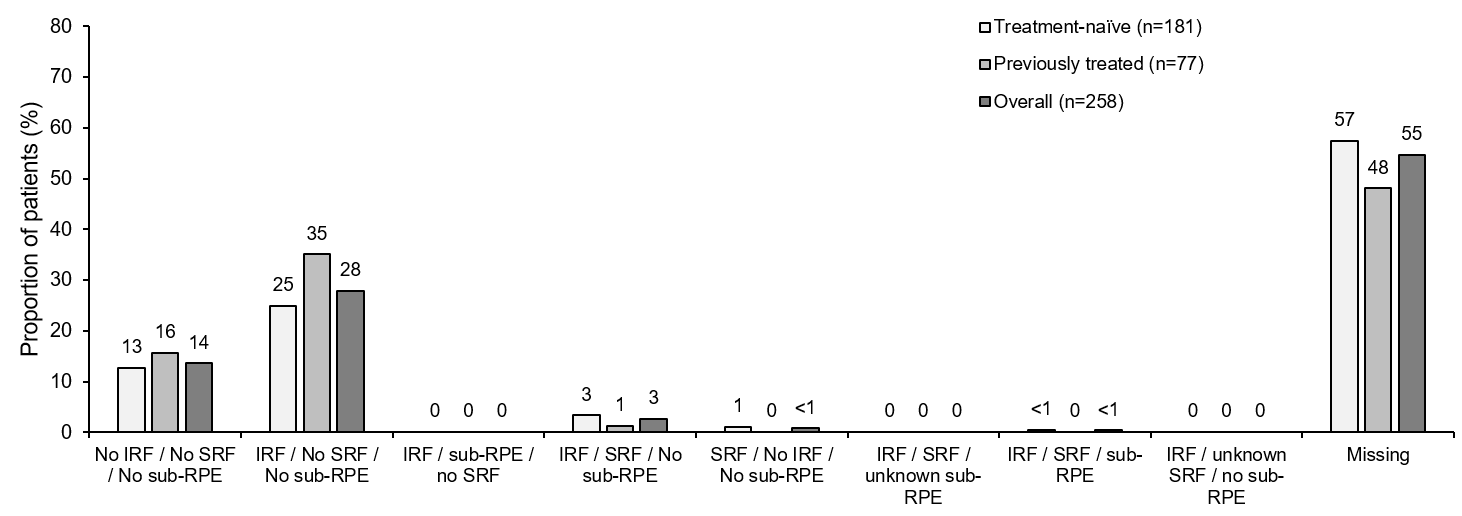


**c**


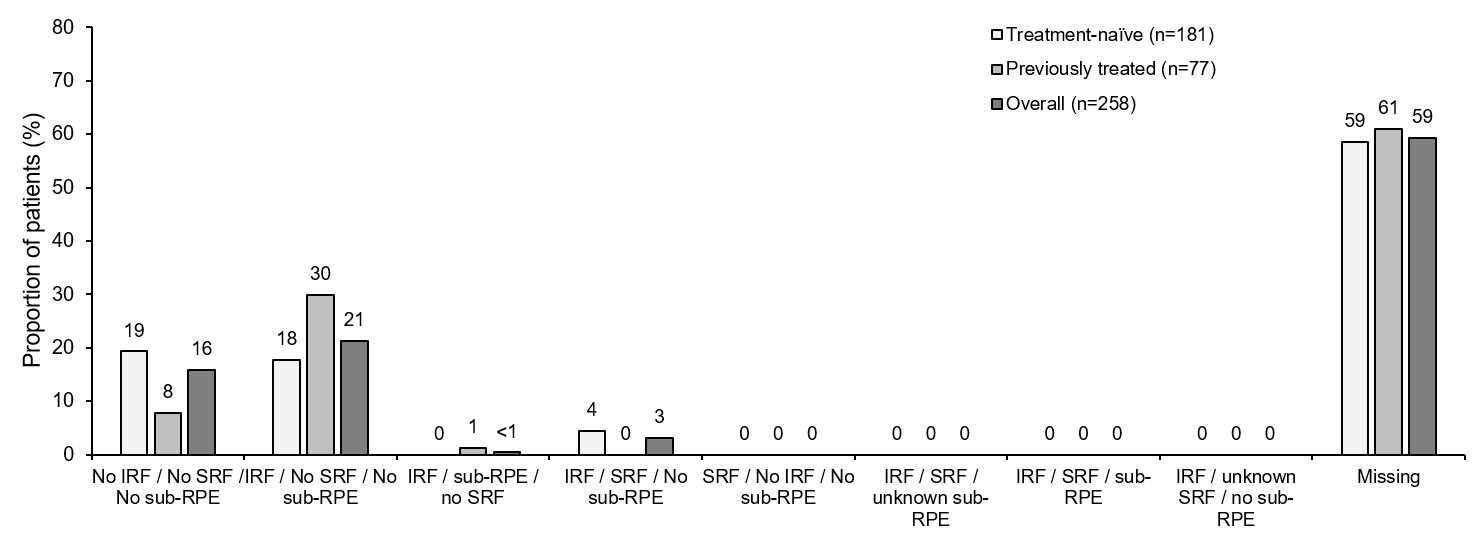

IRF, intraretinal fluid; RPE, retinal pigment epithelium; SRF, subretinal fluid.

# Appendix. List of participating investigators and clinics.

**Argentina:** Marcelo Reinhart (Olavarría); Gastón Gómez Caride (Quilmes); Herminio Negri (Ciudad Autonoma de Buenos Aires); Gerónimo Galván (La Plata, Buenos Aires); Juan Irungaray (Lanus Oeste); Mariano Irós (Córdoba); Matko Vidosevich (Rosario); Noe Rivero (Villa Gdor. Galvez); Tamara Zompa (Ciudad Autonoma de Buenos Aires); Juan Pablo Francos (Ciudad Autonoma de Buenos Aires); Paula Salgado (Ciudad Autonoma de Buenos Aires); Gerardo Caceres Barrios (Escobar); Octavio Regnasco (Escobar); **Colombia**: Francisco J. Rodríguez (Bogotá); Hildegard Piñeros (Barranquilla); Juan Arias (Bucaramanga); Javier Buendia (Medellín); Gustavo Adolfo Navarro Naranjo (Popayan); Beatriz Endo (Cali); Myrian Hernandez (Manizales); **Costa Rica:** Lihteh Wu (San José), Teodoro Evans Tinoco (San José); **Mexico:** Gerardo Garcia (Ciudad de México); Andres Padilla (Guadalajara); Adriana Gómez Cespedes (Chihuahua); Jose Dalma (Ciudad de México); Rene Cano (Ciudad de México); Jans Fromow-Guerra (Ciudad de México); Natalia Saldaña (Ciudad de México); Juan Manuel Jimenez (Ciudad de México); Renata del Carmen García Franco (Queretaro); Adriana Solis Vivanco (Mexico D.F.); Angeles Yael Hernandez Vazquez (Cuatitlan Izcalli).
